# Availability and Rational Use of Drugs in Primary Healthcare Facilities Following the National Drug Policy of 1982: Is Bangladesh on Right Track?

**DOI:** 10.3329/jhpn.v30i1.11289

**Published:** 2012-03

**Authors:** Syed Masud Ahmed, Qazi Shafayetul Islam

**Affiliations:** Research and Evaluation Division, BRAC, BRAC Centre, 75 Mohakhali, Dhaka 1212, Bangladesh

**Keywords:** Costs and cost analysis, Drug-use, Drugs, Health expenditure, National drug policy, Bangladesh

## Abstract

In Bangladesh, the National Drug Policy (NDP) 1982 was instrumental in improving the supply of essential drugs of quality at an affordable price, especially in the early years. However, over time, evidence showed that the situation deteriorated in terms of both availability of essential drugs and their rational use. The study examined the current status of the outcome of the NDP objectives in terms of the availability and rational use of drugs in the primary healthcare (PHC) facilities in Bangladesh, including affordability by consumers. The study covered a random sample (n=30) of rural Upazila Health Complexes (UHCs) and a convenient sample (n=20) of urban clinics (UCs) in the Dhaka metropolitan area. Observations on prescribing and dispensing practices were made, and exit-interviews with patients and their attendants, and a mini-market survey were conducted to collect data on the core drug-use indicators of the World Health Organization from the health facilities. The findings revealed that the availability of essential drugs for common illnesses was poor, varying from 6% in the UHCs to 15% in the UCs. The number of drugs dispensed out of the total number of drugs prescribed was higher in the UHCs (76%) than in the UCs (44%). The dispensed drugs were not labelled properly, although >70% of patients/care-givers (n=1,496) reported to have understood the dosage schedule. The copy of the list of essential drugs was available in 55% and 47% of the UCs and UHCs respectively, with around two-thirds of the drugs being prescribed from the list. Polypharmacy was higher in the UCs (46%) than in the UHCs (33%). An antibiotic was prescribed in 44% of encounters (n=1,496), more frequently for fever (36-40%) and common cold (26-34%) than for lower respiratory tract infection, including pneumonia (10-20%). The prices of key essential drugs differed widely by brands (500% or more), seriously compromising the affordability of the poor people. Thus, the availability and rational use of drugs and the affordability of the poor people have remained to be achieved in Bangladesh even 27 years after approving the much-acclaimed NDP 1982.

## INTRODUCTION

The availability of essential drugs (medicines considered indispensable for the treatment of a disease) and the affordability of the common people are crucial for the successful functioning of any health system (1). It is also an important factor to prevent bypassing of primary healthcare (PHC) facilities by the common people (2). In Bangladesh, the National Drug Policy (NDP) 1982 was instrumental in improving the supply of essential drugs of quality at an affordable price, especially in the early years (3). An essential drugs list (EDL), approved by the Government, initially identified 150 drugs with controlled prices. Due to the policy of buying raw materials from international competitive markets under the new policy, the prices of essential drugs fell sharply in the subsequent years. During 1981-1991, the retail prices of drugs increased by 20% in the local market. The pharmaceutical industry in Bangladesh also developed rapidly following the implementation of the NDP 1982 (4). In 1980, eight multinational companies manufactured 75% of all products (by value) while indigenous pharmaceuticals now claim a market share of more than 75% (5).

Evidence showed that essential drugs were not often available, especially in the government health facilities (6). Besides, the irrational use of drugs, such as over-prescribing, prescribing of multiple drugs, use of unnecessary expensive drugs, and overuse of antibiotics and injections were observed (3,7). The number of regulated drugs was reduced to 117 in 1993 and again increased to 209 in 2007 to reflect advancement in medical sciences (8), with loosening of control in fixing the prices of non-essential drugs in particular and all drugs in general (9).

The Central Medical Store of the Government distributes drugs in the public hospitals and facilities (10). The retail distribution presents a totally chaotic situation without any regulatory mechanism. Although the persons dispensing drugs at the retail shops should have at least a short training of eight weeks before applying for a license, in practice, this is hardly followed. Of 200,000 drug stores that sell drugs over-the-counter in the country, only 76,000 (38%) are approved (licensed) by the Government (11).

The retail drug shops are often the first and the only source of healthcare outside home for the majority of patients in developing countries (12), and Bangladesh is no exception to this. Anybody can buy any drug in any quantity, including addicting drugs, from these shops without any prescription. Bangladesh is one of the few countries where there is high out-of-pocket (OOP) expenditure on drugs by households, which amounts to around 70% of the total OOP expenditure on health (13). Aggressive marketing by the pharmaceutical companies in Bangladesh and the free availability of ‘prescription only’ drugs in the unlicensed and unregulated retail outlets (drug shops) have worsened the situation for the rational use of drugs (14,15).

The meagre resources of the Directorate of Drug Administration (the supreme regulatory authority in the country for drug-related affairs) have made it practically impossible to monitor and regulate these drug stores, including the large pharmaceutical sector in the country (5). As a result, many drugs enter the market without proper quality-assessment procedures, and the market is flooded with counterfeit, substandard and expired drugs (16). For example, in a testing by the drug regulating authority, 69% of paracetamol tablets and 80% of ampicillin capsules, manufactured by small companies, were found to be below the acceptable standard, and results of an assay of drugs involving 15 brands of ciprofloxacin showed that 47% of collected samples contained less-than-required ingredients (17).

In the public sector, primary-level healthcare in Bangladesh consists of Upazila Health Complexes (UHCs), with inpatient (31-bed) and basic laboratory facilities, and a network of sub-centres and community health workers. Besides the shortage of skilled health workforce, these centres also lack appropriate diagnostic facilities and medicines, causing a gradual decline in the use of government health services for the treatment of diseases from 17% in 2000 to 13% in 2003 (18). It was observed that diagnoses made at the PHC level were mainly based on presenting complaints, history-taking, and clinical examinations (19).

There has been no comprehensive study of the NDP in Bangladesh, which requires investigation of the state of the indicators relating to structure (pharmaceutical system's capacity to achieve the stated goals), process (the degree to which necessary activities are carried out and progress over time) and outcome (to assess availability, affordability, quality, and rational use) (20). A baseline survey, conducted in 1992 on the use of drugs at some public-sector PHC facilities in rural Bangladesh, found that the availability and use of essential drugs were very low and irrational, and over-prescribing of drugs was common (7). Since then, no study has been conducted to track the changes over the years and guide practitioners. This study aimed at fulfilling this knowledge gap by investigating the availability and rational use of drugs and the affordability of the common people in both rural and urban PHC facilities in the country. This is expected to help the policy-makers/practitioners understand the present situation and take remedial measures to reach the poor with ‘quality drugs at low cost’ (21). Besides, responsiveness of the PHC facilities and client satisfaction were also explored.

## MATERIALS AND METHODS

Data for the study originated from a nationwide survey in 2009 on the rational use of essential drugs in the PHC facilities (22). The study was designed as a facility-based cross-sectional survey to collect data on core drug-use indicators as recommended by the World Health Organization (WHO) which could be implemented “by individuals without special training or access to many resources” (23).

### Sampling

Due to the time and resource constraints, the sample was limited to 30 rural UHCs and 20 UCs in the Dhaka City Corporation (DCC) area. The 30 UHCs were randomly selected from the six divisions proportionate to size, i.e. the division with the largest number of UHCs provided the largest proportion of 30 UHCs in the sample. Again, two UCs in the DCC area were selected at random from each of the 10 zones where the Urban PHC Project II provides outpatient services under a partnership agreement with non-government organizations (NGOs) selected through competitive bidding (24). The list of the UCs was provided by the NGOs in their respective working areas.

Thirty patients attending the outpatient departments of each of the UHCs and UCs for common acute illnesses were enrolled in the study. They were selected from the total number of patients visiting the PHC facility in working days (consecutive) until the required number of patients was obtained. Patients were selected by systematic random sampling to avoid bias from timing of the survey (rush hours in the beginning or end of clinic sessions) or freshness or fatigue of the healthcare providers/workers. Thus, 1,500 patients—900 in the UHCs and 600 in the UCs—were included in the study.

### Techniques and tools

Data were collected through observations on prescribing and dispensing practices and recording information, exit-interviews with patients and their attendants (for understanding dosage, responsiveness of the system, and satisfaction with services), and a mini-market survey (to elicit prices of essential drugs for common illnesses). A pretested structured form was used for recording relevant data from patient-provider interactions. Another pretested, semi-structured questionnaire was used for recording information from exit-interviews of patients or attendants. Finally, a checklist was used for recording price-related information. The data-collection instruments were prepared based on literature review and peer experience. These were then pretested by a core group of interviewers who received intensive training and orientation from the researchers conducted in an area outside the study areas.

Based on the Government-approved EDL, a reference list of key essential drugs for common illnesses (henceforth reference list), selected from reality check of patient registers at the UHCs, was prepared (8). This reference list of 20 drugs was used for checking the availability of drugs in the PHC facilities and also for collecting information on prices through the mini-market survey.

### The survey

Trained interviewers collected data during February-March 2009. To understand the prescribing and dispensing practices, the selected UHC/UC was observed for two consecutive days during usual office hours (9 am-1:30 pm). The two-member survey team started the day by taking permission from the chief executive of the UHC/UC to proceed with the study, exploring whether there was an EDL (posted in public or in file) in the facility and going through the records of the past seven days to get an idea about the daily average patient-load of the facility. The latter information was used for deciding upon the interval required for taking the systematic random sample of 30 patients.

One interviewer placed himself at the door of the Doctor's Chamber and recorded the time of entry and exit of the sampled patient using a stopwatch. The prescribing indicators were recorded from the prescription slip immediately after the doctor-patient interaction was over. Another interviewer, posted near the dispensary, followed the sampled patient when s/he came out of the doctor's chamber. The time of submitting the prescription slip to the dispenser and the time when drugs were dispensed were recorded, and the dispensing time was calculated. The number of drugs in the prescription slip, the number of drugs served by the dispenser, and labelling of the drugs served were recorded. Also, for confirming the availability of drugs, the reference list of drugs was read in front of the storekeeper/dispenser, and he was asked to show the drugs, if available. When the drug was shown, only then it was recorded as available.

The same interviewer conducted an exit-interview with the patient or his/her attendant to elicit information on their understanding of dosage, responsiveness of the system, and satisfaction with services. The interview was conducted in a place away from the prescribing and dispensing site but within the premises of the facility. The whole process continued in this cycle until the desired number of patients was enrolled.

Finally, a mini-market survey was conducted in the rural areas to find the extent of variation in market prices of essential drugs for common illnesses. Near each UHC in the sample, 10 drug stores were randomly chosen, and the minimum and maximum prices of drugs included in the reference list were recorded. The prices were obtained from the sales people at the drug stores. The cost of a full-course of drugs for three common illnesses among the study subjects was reviewed to understand the affordability of the extreme poor people.

### Management and analysis of data

The SPSS PC+ software (version 12) was used for analyzing data. The summary statistics of the different drug-use indicators were compiled to compare the rural and urban facilities (UHCs and UCs in the DCC area respectively).

### Ethical clearance

The study proposal passed through the usual institutional review process at the Research and Evaluation Division of BRAC and the ethical review board of the James P. Grant School of Public Health, BRAC University. No invasive procedure was done. Informed verbal consents were obtained from the respondents who were largely illiterate or semi-literate. The written consent form was read out and explained to the respondents. When the investigator was satisfied that the respondent understood it, including its implications, and had agreed to participate, only then s/he was included in the survey. Anonymity of the respondents was maintained at all stages of analysis of data.

## RESULTS

In total, 900 patient-provider encounters in the UHCs and 600 patient-provider encounters in the UCs (5 patients declined to be interviewed) were observed. Around 27% and 40% of the patients attending the UHCs and UCs respectively were aged ≤5 years while the proportion of patients aged ≥60 years attending the UHCs was twice (7.2%) that of the UCs (3.4%). In both the types of facilities, more women sought care than men.

### Availability of drugs

Forty-seven percent of the UHCs and 55% of the UCs had a copy of the EDL (Table 1). None of the facilities had all the 20 drugs included in the reference list of key essential drugs for common illnesses. However, 6% of the UHCs and 15% of the UCs had at least 15 of the 20 drugs in the reference list.

### Rational use of drugs

The average number of drugs prescribed per visit (prescription) was 2.2 in the UHCs and 2.5 in the UCs (Table 1). The proportion of encounters (n=1,496) with an antibiotic prescribed was around 44%. In more than 60% of the encounters, drugs were prescribed from the EDL. Polypharmacy or prescribing three or more drugs was quite common, especially in the UCs (46% opposed to 33% in the UHCs) (Table 2).

**Table 1.** Availability and use of drugs by study areas (%)

Characteristics	Rural UHCs (n=900)	UCs in DCC area (n=596)*
Availability		
Facilities having a copy of the list of essential drugs	47.0	55.0
Facilities where at least 15 key essential drugs from the list of essential drugs are available	6.0	15.0
Use		
Average number of drugs per encounter	2.2	2.5
Of encounters with an antibiotic prescribed	45.0	42.7
Of drugs prescribed from the list of essential drugs	63.0	66.1

*Four patients had incomplete data and excluded from analysis;

DCC=Dhaka City Corporation;

UCs=Urban clinics;

UHCs=Upazila Health Complexes

Table 3 shows the extent of prescribing antibiotics for selected common illnesses (exclusive) by the two types of facilities. A greater tendency was observed to prescribe antibiotics for fever (40% in the UHCs and 36% in the UCs) and common cold/cough (26% in the UHCs and 34% in the UCs) than for acute respiratory infection (ARI) (including pneumonia) (10% in the UHCs and 19% in the UCs). Thus, the UCs performed relatively rational when prescribing antibiotics. In all the cases, antibiotics were prescribed without any laboratory investigation.

### Variation in prices of drugs

A wide variation was observed in the lowest and the highest market prices of the same drug. The differences were sometimes more than 500%, e.g. tablet iron-folic acid–1,650%, tablet mebendazole–900%, and benzyl benzoate lotion–817% (Table 4).

Table 5 shows the cost of drugs for a standard regimen for the treatment of three common illnesses, such as hyperacidity (including peptic ulcer), amoebic dysentery, and acute respiratory tract infection (including pneumonia). The cost varied from 7% to 8% of weekly income of the extreme poor for treating hyperacidity and amoebic dysentery to 20% for treating ARI with antibiotics.

### Responsiveness of the system and client satisfaction

The average consulting and dispensing time was less in the UHCs (1-2 minutes) than in the UCs (2-6 minutes) (Table 6). The proportion of drugs dispensed out of those prescribed was much higher in the UHCs (76%) than in the UCs (44%). Only 65% of the drugs dispensed in the UHCs and 43% in the UCs were labelled (we considered drugs as labelled if these could be identified by either the inscription on its body or the name printed in the original package disbursed). While instructions on how to take drugs were given verbally, more than 70% of the patients or care-givers reported that they understood how to take the dispensed drugs.

**Table 2.** Polypharmacy by study areas (%)

Prescription containing the number of drugs	Rural UHCs (n=900)	UCs in DCC area (n=596)*
1	18.1	14.8
2	49.0	38.9
3	27.0	30.0
4 or more	5.9	16.2

*Four patients had incomplete data and excluded from analysis;

DCC=Dhaka City Corporation;

UCs=Urban clinics;

UHCs=Upazila Health Complexes

**Table 3.** Antibiotics prescribed for selected common illnesses by study areas (%)

Illnesses for which antibiotics were prescribed	Rural UHCs (n=405)	UCs in DCC area (n=255)
Fever	40.5	36.0
Cough/common cold	26.0	34.0
Lower respiratory tract infection (including pneumonia)	10.0	19.0
Diarrhoea	4.0	5.0
Dysentery	4.4	6.3
Body ache	7.5	7.0
Hyperacidity	2.1	1.2
Weakness	2.3	4.0

DCC=Dhaka City Corporation;

UCs=Urban clinics;

UHCs=Upazila Health Complexes

Table 7 presents the findings of exit-interviews with the respondents, i.e. patients or their attendants. The mean waiting time at the UHCs (17 minutes) was reportedly less than that in the UCs (24 minutes). The UHCs performed poorly in terms of physical examinations (42%) and maintenance of privacy (34%) compared to the UCs (76% and 66%. respectively). A negligible proportion of the respondents from the UHCs reported paying unofficial charges. Overall, the level of satisfaction with services received from the UCs was quite high (95% compared to 84% for the UHCs). This was further corroborated by the fact that most (>90%) respondents mentioned they would suggest their friends/relatives to visit these facilities for seeking care.

## DISCUSSION

The study was conducted to explore the current state of the availability and rational use of drugs and the affordability of the poor people in rural and urban Bangladesh. We used the core indicators of WHO to investigate drug-use in PHC facilities (25). The findings of the study revealed that the availability of key essential drugs for common illnesses was poor, and an unacceptable level of polypharmacy has been practised in the PHC facilities, including misuse and overuse of antibiotics. Generic prescribing was totally non-existent, and the affordability of the common people was severely compromised due to the variation in the retail prices of key essential drugs as much as 500% or more. The implications of these findings are discussed in the context of ‘reaching the poor with quality drugs at low cost’ as envisioned in the NDP 1982.

The EDL includes a core list of minimum drugs that satisfy the healthcare needs of the majority of the population in a particular country and should be available all times in adequate quantity and prescribed in appropriate dosage (20). While comparing with the previous survey of UHCs (7), an improvement was observed in relation to the availability of a copy of the EDL but deterioration was observed in the availability of key essential drugs for common illnesses. This is also consistent with the fact that lack of medicines was the most common complaint for which progressive deterioration in the use of government health services was observed during 1999-2003 (18).

Around two-thirds of the drugs were prescribed from the EDL. The drugs prescribed from the EDL in the UHCs recorded a substantial fall compared to what was found by Guyon *et al*. (from 85% to 63% in this study) (7). A similar level of prescription from the EDL was reported from Serbia (25) but a higher level was reported from India (26), Laos (27), and Tanzania (28). Thus, Bangladesh is lagging behind other low-income countries in this aspect. None of the drugs dispensed from any of the facilities was labelled properly (i.e. generic name of drug, dosage, etc.) as also observed in India (26), Tanzania (28), and Cambodia (29). However, the self-reported knowledge of patients or attendants on correct dosage observed in the UHCs (around 65%) was comparable (26,27) or even better than that reported in other studies (28,29).

**Table 4.** Lowest and highest prices of selected essential drugs for common illnesses

Reference drugs from the list prepared by the study team (unit)		Current market price (Tk*)		% of difference
		Lowest	Highest	
1	ORS (1 pack)	2.00	5.00	150
2	Tablet co-trimoxazole (1 tablet)	0.80	2.50	213
3	Syrup co-trimoxazole (60 mL)	11.00	26.65	142
4	Syrup amoxicillin (100 mL)	20.00	60.00	200
5	Tablet ciprocine (1 tablet)	4.00	16.00	300
6	Syrup ciprocine (100 mL)	37.00	95.00	157
7	Tablet aluminium hydroxide + magnesium hydroxide (1 tablet)	0.50	1.50	200
8	Tablet aluminum hydroxide + magnesium hydroxide (1 tablet)	11.00	65.00	491
9	Tablet ranitidine 150 mg (1 tablet)	1.00	4.00	300
10	Tablet paracetamol (500 mg) (1 tablet)	0.50	2.00	300
11	Tablet aspirin (300 mg) (1 tablet)	0.50	2.00	300
12	Tablet IFA (1 tablet)	0.20	3.50	1650
13	Tablet B complex per bottle (45 tablets)	12.00	90.00	650
14	Tablet ascorbic acid (1 tablet)	0.50	2.00	300
15	Tablet mebendazole (1 tablet)	0.50	5.00	900
16	Tablet albendazole (1 tablet)	1.00	6.00	500
17	Tablet atenolol 50 mg (1 tablet)	0.70	4.00	330
18	Tablet prednisolone 5 mg (1 tablet)	0.50	1.50	200
19	Benzyl benzoate lotion 25% (1 ph)	6.00	55.00	817
20	Chloramphenicol eye drop 10 mL (1 bottle)	10.00	35.00	250
21	Chloramphenicol eye ointment (1 tube)	7.00	45.00	543
22	Xylometazoline nasal drop 15 mL (1 bottle)	6.00	35.00	483
23	Miconazole ointment 10 g (1 tube)	13.00	90.00	592
24	Tablet metronidazole 400 mg (1 tablet)	0.50	3.00	500

*Exchange rate at the time of survey: US$ 1=Tk 69;

IFA=Iron-folic acid;

ORS=Oral rehydration solution

Polypharmacy or prescribing three or more drugs increases the risk of drug interactions, dispensing errors, and proper comprehension of the correct dosage (25). In this study, we found polypharmacy on the rise: compared to 5% polypharmacy reported earlier in UHCs (7), we found this to be 33%, which should raise our concern. The proportion of polypharmacy was even higher (46%) in our UCs compared to that found in Indonesia (30). The average number of drugs prescribed per encounter in the UHCs (2.2) was higher than that observed in the earlier study (1.4). Such a higher number was also observed in studies in Serbia (25), India (26), Laos (27), and Tanzania (28).

The proportion of encounters/prescriptions with an antibiotic prescribed was around 44% when UHCs and UCs taken together; it was higher in the UHCs (45%) compared to an earlier study in Bangladesh (25%) (7) and other studies (27,30). Of more concern is the fact that antibiotics were much less frequently used for lower respiratory tract infections and pneumonia where it is warranted. Such an irrational use of antibiotics is responsible for the development of antibiotic resistance (31).

The anarchy prevailing in the drug market is well-amplified by the wide variation in the prices of drugs by brands; sometimes the difference ranged from 500% to more than 1,000%. A similar situation was also observed in other low and middle-income countries (32). This seriously compromises the affordability of the poor people for quality drugs. Lower-priced drugs are mainly marketed by small local companies against whom complaints of producing low-quality drugs are common (33). As observed in the present study, the cost of a standard regimen of treatment for a common illness was quite substantial for the extreme poor when compared with their income of one dollar a day. The poor regulatory and supervisory mechanism in the country contributes to this price-hike (9,21). This high cost of medicines and the low availability of essential medicines due to the failure of the government regulatory mechanism were also observed in middle-income countries, such as Malaysia, with the relatively-stable and effective public-health system (34). Evidence shows that it is possible to improve the availability of essential drugs, including the affordability of consumers by market regulation throughout the supply-chain from the manufacturer to the patient and through the competitive purchasing process of raw materials (35,36). As OOP expenditure for drugs is high in Bangladesh and all prescribed drugs are not always available at facilities, the poor regulation contributes to the catastrophic health expenditure for poor households (18,37).

**Table 5.** Cost of standard minimum dose of drugs commonly prescribed by upazila doctors for selected common illnesses as a percentage of weekly income of the extreme poor (@ US$ 1 a day)*

Illness/disease	Drug	Standard minimum dose commonly prescribed by upazila doctors	Cost (Tk) (US$ 1=Tk 69)*	Cost as percentage of weekly income**
Hyperacidity, including peptic ulcer	Aluminium hydroxide+ magnesium hydroxide suspension	15-30 mL everyday up to 6 weeks or until pain subsides	55 (per week)	13.28
	Tab ranitidine 150 mg	2 tablets daily for 4 weeks	28 (per week)	6.76
Amoebic dysentery	Tablet metronidazole 400 mg	800 mg 3 times daily for 5 days (adult)	30	7.24
	Syrup metronidazole	15 mL daily for 5 days (child)	45	10.86
Lower respiratory tract infection, including pneumonia	Capsule amoxicillin	500 mg 3 times daily for 7 days	105	25.36
	Syrup amoxicillin	10 mL 3 times daily for 7 days (children aged >2 years)	90	21.73
	Syrup co-trimoxazole	10 mL daily for 7 days (children aged up to 5 years)	40	9.66

*Exchange rate at the time of survey;

**Assuming work for 6 days a week

**Table 6.** Patient-care indicators by study areas

Indicator	Rural UHCs (n=900)	UCs in DCC area (n=596)*
Average consultation time (minutes)	1.8	5.8
Average dispensing time (minutes)	0.9	2.1
% of drugs actually dispensed	76.3	44.0
% of drugs adequately labelled	65.4	43.0
% of patient's knowledge of correct dosage (self-reported)	73.0	76.0

*Four patients had incomplete data and excluded from analysis;

DCC=Dhaka City Corporation;

UCs=Urban clinics;

UHCs=Upazila Health Complexes

Responsiveness or responding to people's expectations is one of the main objectives of the health system (38). In the core indicators of WHO for the rational use of drugs, the quality of care provided is measured by the time taken in consultation and dispensing (including time for instruction on how to take drugs). The average consulting and dispensing time in the UHCs increased from what was found earlier (15) but lagged behind other countries, e.g. Serbia (25). Such a short time is neither adequate for history-taking and examination of patients nor giving them sufficient information on the dosage of drugs and necessity for compliance. Also, lack of proper physical examinations of patients and maintenance of privacy question the responsiveness of the system.

**Table 7.** Findings from exit-interviews on satisfaction with services received, by sex and study areas (%)

Characteristics of services received	Rural UHCs			UCs in DCC area		
	Male (n=379)	Female (n=521)	All (n=900)	Male (n=189)	Female (n=406)	All (n=595)*
Waiting time at facilities (mean)	14.6	18.6	16.9	23.9	24.6	24.5
Doctors listened to problems attentively	94.0	94.0	94.0	98.0	99.0	99.0
Physical examinations done by doctors	44.0	40.0	42.0	76.0	76.0	76.0
Privacy was maintained by doctors	35.0	33.0	34.0	62.0	68.0	66.0
All prescribed medicines were given from the facility	65.0	73.0	70.0	29.0	37.0	34.0
Paid unofficial charge(s)	4.0	3.0	3.0	0.0	0.0	0.0
Satisfied with services	84.0	83.0	84.0	95.0	95.0	95.0
Will suggest to relatives/friends to visit this facility	93.0	93.0	93.0	99.5	98.0	99.0

*Five patients refused to participate;

DCC=Dhaka City Corporation;

UCs=Urban clinics;

UHCs=Upazila Health Complexes

This study is an improvement from the earlier study on the use of drugs at the UHCs (7). It covered a larger, representative sample of upazilas in the country and additionally included an urban sample from the DCC area. The sample-size was adequate as per recommendations of the International Network for the Rational Use of Drugs and WHO to compare facilities (23). On the other hand, due to practical reasons, we had to use proxy indicators for certain components, such as ‘labelling’, and we did not explore whether drugs prescribed followed the standard guidelines as most recorded diagnoses were non-specific.

### Conclusions

Except the early years in the eighties, the NDP 1982 could not ensure the availability and rational use of drugs and the affordability of the common people over the long term in Bangladesh as exemplified in the present study. The situation has rather deteriorated in the past 15 years, especially in rural areas. Concerted efforts are needed to motivate and train those in the medical profession and allied health professions (e.g. nurses, paramedics, informal allopathic practitioners, drug dispensers, and manufacturers) about the benefits of generic prescribing (which can lead to cost savings) and prescribing from the national EDL, especially for the poor. Polypharmacy and overuse/misuse of drugs, especially antibiotics, should be discouraged to avoid drug resistance and its consequences. Besides, strengthening the regulatory capacity of the Directorate of Drug Administration for quality and price will be needed. Finally, the need of essential laboratory services at the PHC-level facilities for the proper diagnosis of illnesses which require antibiotics (e.g. childhood pneumonia) and the rational use of drugs cannot be overemphasized (39).

## ACKNOWLEDGEMENTS

The study was funded by the Swedish International Development Agency through the Bangladesh Health Watch, a civil society initiative “to regularly and systematically measure and monitor the country's progress and performance in health.”

The authors gratefully acknowledge the time and experiences shared by the staff of the participating UHCs and UCs in the study. Thanks are due to Dr. Abbas Bhuiya of icddr,b and Dr. Mushtaque R. Choudhury of the Rockefeller Foundation for their encouragement and guidance in conducting the study, and Prof. Md. Sayedur Rahman of the Bangabandhu Sheikh Mujib Medical University for helping in designing the study. Special thanks are due to Mr. Farid Ahmed of the Research and Evaluation Division, BRAC, for helping with data and field management and Mr. Hasan Shareef Ahmed for editing the manuscript.
